# Rhabdomyolysis Induced by Rhinovirus: A Case Report

**DOI:** 10.7759/cureus.22784

**Published:** 2022-03-02

**Authors:** Rawia F Albar, Haitham A Alasmari, Sultan A Neazy, Abdullah S Alzahrani, Kenan Nejaim, Dareen A Qattan

**Affiliations:** 1 Pediatrics, King Abdulaziz Medical City, Jeddah, SAU; 2 College of Medicine, King Saud Bin Abdulaziz University for Health Sciences, Jeddah, SAU

**Keywords:** case report, creatine kinase, viral-induced rhabdomyolysis, rhinovirus, rhabdomyolysis

## Abstract

The occurrence of rhabdomyolysis in pediatric patients is considered a rare complication that can follow certain viral infections in a syndrome better defined as virus-associated rhabdomyolysis. In this research, we will present the case of a ten-year-old male patient who presented to the emergency department with chief complaints of severe bilateral leg pain and inability to walk. Furthermore, the patient complained of dysphagia for both solid and liquid along with dark-colored urine. Initial investigations showed an increase in creatine kinase (CK), C-reactive protein (CRP), and liver enzymes. Additionally, urine analysis was obtained with positive traces of blood, protein, and white blood cell. X-ray was ordered with no significant finding. Finally, the diagnosis was reached in accordance to the results of the respiratory panel multiplex (PCR) as the third case of rhinovirus-induced rhabdomyolysis. He was treated with isotonic intravenous fluids, and he was discharged on hospital day 20 with a CK of 2062 IU/L. The patient was discharged fully recovered, was able to stand and walk alone, and with no complications. In this third to be reported case of rhinovirus-induced rhabdomyolysis, we aim to increase the knowledge among the general pediatric field regarding the possible presentation and treatment of any similar case.

## Introduction

Human rhinovirus (HRV) is a member of the Picornaviridae family and the genus Enterovirus [1]. HRV is a positive-sense, single-stranded-RNA (ssRNA) virus [1]. It is known as the most common causative agent of respiratory viral illnesses during spring, summer, and fall. As for the influenza virus and respiratory syncytial virus (RSV), they predominate in winter [2]. HRV can present either as an asymptomatic infection or symptomatic with a presentation that varies as an upper or lower respiratory infection [3]. Moreover, it can cause extrapulmonary complications including pulmonary edema, diabetic ketoacidosis, and hyperosmolar coma [3]. There are only two reported cases about the relationship between HRV infections and rhabdomyolysis [4,5]. Although it is a rare case to encounter, it is potentially life-threatening. Hence, in this case report, we aim to present a case about a young male diagnosed with rhabdomyolysis that was induced by an HRV infection expectantly increasing the awareness of rhabdomyolysis presentation and its management among the medical community.

## Case presentation

A ten-year-old Saudi male patient presented to the emergency department (ED) with severe bilateral leg pain and an inability to walk for three days. Ten days prior to the presentation, the patient complained of rhinorrhea, cough, severe right leg pain and swelling that started after a long period of running. In addition, it was accompanied by dark-colored urine. Three days later, he started to have the same presentation in his left leg. He also complained of mild difficulty in swallowing solids and liquids, with no history of choking. Upon arrival to the ED, the patient looked very sick, coughing, crying from pain, tenderness all over his body, and was unable to walk. He was evaluated and found to be tachycardic (heart rate 120 bpm), afebrile (36.5°C), normotensive (121/70 mmHg), and had normal oxygen saturation (100% on room air). On physical examination, he was conscious, alert, and oriented. The patient had decreased power in the lower limbs and trunk (3/5) since rhabdomyolysis cause generalized weakness. Also, he was unable to move both of his legs. Besides, there was no history of any drug ingestion. The patient presented in the ER with high creatine kinase (CK), C-reactive protein (CRP), and liver enzymes. CK was elevated at 35892 IU/L, CRP was 24.5 mg/L, creatinine was 33 (umol/L), aspartate aminotransferase (AST) was 913 IU/L, and alanine transaminase (ALT) was 869 U/L. In addition, normal renal function with no sign of acute kidney injury. Furthermore, urine analysis was indicating hematuria and positive myoglobin. Additionally, rheumatology serology labs were negative apart from antinuclear antibodies (ANA). Moreover, ultrasound was negative and excluded deep venous thrombosis (DVT). Also, the nerve conduction study was normal and there was no neurogenic cause. Of the most importance, respiratory panel multiplex (PCR) was done and it excluded covid-19 and other viral infections. However, it revealed that he was positive for human rhinovirus infection. Therefore, the diagnosis was made as rhinovirus-induced rhabdomyolysis. Due to the patient’s high CK and transaminitis, he was admitted under the general pediatric ward and was started on intravenous fluids 500ml normal saline. The patient had moderate chest pain that increased with inspiration and pulmonary embolism was ruled out by computerized tomography. After that, he was given acetaminophen as needed until his symptoms subsided. The patient continued to receive intravenous fluid resuscitation, and his CK levels, AST, and ALT continued to improve, as shown in Table 1.

**Table 1:** Laboratory results throughout hospitalization days ALT: Alanine transaminase; AST: Aspartate aminotransferase.

	Day 1	Day 2	Day 3	Day 4	Day 5	Day 6	Day 7	Day 8	Day 9	Day 10	Day 11	Day 12	Day 13	Day 14	Day 15	Day 16	Day 17	Day 18	Day 19	Day 20
Creatinine kinase U/L	35892	19890	15479	23644	26157	19551	18137	13059	10495	14750	14050	11408	14247	11715	9444	7307	5104	4790	3163	2062
ALT IU/L	869	755	699	775	869	819	911	751	751	761	761	761	761	761	686	686	686	686	396	396
AST U/L	913	621	455	560	680	509	524	404	404	544	544	544	544	544	259	259	259	259	131	131

## Discussion

Rhabdomyolysis is a syndrome that is characterized by the breakdown of skeletal muscle fibers, resulting in a subsequent release of intracellular contents into the circulatory system [6]. Thus, an increase of the levels of cell content in the blood such as CK, glutamic oxalacetic transaminase, lactate dehydrogenase, aldolase, the haeme pigment myoglobin, potassium, phosphates, and purines [6]. However, rhabdomyolysis is clinically defined as muscle symptoms with serum CK of more than 10 times the upper limit of the normal value, with a creatinine elevation consistent with pigment nephropathy and usually with brown urine with myoglobinuria [7]. Rhabdomyolysis in the pediatric population can be caused by any kind of muscle damage, such as traumatic injuries, connective tissue or metabolic disorders, exercise, drug overdose, or exposure to toxins [8]. In addition, infections have been reported to be associated with rhabdomyolysis, such as influenza and severe acute respiratory syndromes [9,10]. Also, in children below nine years, viral infection is the most common cause [11]. A case report published in 2016, described the association of human rhinovirus with rhabdomyolysis [4]. The patient presented with a cough lasting for a week and fever for one day [4]. It was found that his creatine kinase, alanine transaminase, and aspartate transaminase levels were elevated, and his urine myoglobin was 10,024 µg/L [4]. The patient was managed with intravenous fluid hydration after a diagnosis of rhabdomyolysis was made. On the sixth day, he was weaned off oxygen and was discharged home well with improved musculoskeletal pain [4]. Our patient, on the other hand, presented with severe bilateral leg pain with inability to walk that began 10 days prior to hospital admission; he was also having a mild difficulty swallowing solid and liquid meals. The initial laboratory results showed an increase in CK, CRP, and liver enzymes. Additionally, urine analysis revealed very few WBCs, a large amount of blood, and traces of protein. In terms of treatment, the patient received a hydration bolus of normal saline in the ED as well as maintenance intravenous fluid. Furthermore, acetaminophen was given to him to ease the chest pain. In his follow-up after one month of his discharge, his CK level was decreased to 2062 IU/L, his liver function tests were normal.

## Conclusions

In conclusion, our patient presented with a classical rhabdomyolysis symptoms triad. His initial investigations showed elevated CK, CRP, and liver enzymes. After excluding other causes, PCR test was done and detected positive human rhinovirus. The patient then underwent supportive treatment with fluid and analgesics. Although the incidence of rhabdomyolysis caused by rhinovirus is rare, thorough medical treatment is recommended. Early detection, intravenous hydration, and continuous monitoring should be applied for such cases.
